# Metformin Toxicity Masquerading As Acute Abdomen: A Clinical Reminder of Metformin-Associated Lactic Acidosis and Its Management

**DOI:** 10.7759/cureus.79573

**Published:** 2025-02-24

**Authors:** Kishor Pokharel, Laxman Wagle, Dante A Suffredini

**Affiliations:** 1 Internal Medicine, Ascension Saint Agnes Hospital, Baltimore, USA; 2 Critical Care Medicine, MedStar Washington Hospital Center, Washington DC, USA

**Keywords:** continuous renal replacement therapy, extracorporeal treatment, metformin, metformin induced lactic acidosis, type 2 diabetes mellitus

## Abstract

Metformin is commonly used to manage type 2 diabetes mellitus (T2DM), but it is linked to a rare yet potentially life-threatening complication known as metformin-associated lactic acidosis (MALA). MALA typically occurs in patients with renal impairment, but may also be seen in those with liver disease, heart failure, or other metabolic disturbances. Management is primarily supportive, with aggressive interventions including decontamination and extracorporeal treatments such as continuous renal replacement therapy (CRRT) to reverse acidosis and clear metformin metabolites.

A 72-year-old female with T2DM on metformin presented with severe diffuse abdominal pain, lethargy, and severe metabolic acidosis following a colonoscopy five days earlier, which was complicated by dehydration. Imaging ruled out pneumoperitoneum, and exploratory laparotomy revealed no ischemia or perforation. With suspicion of metformin toxicity, the patient was promptly started on CRRT, leading to rapid improvement. A metformin level confirmed the diagnosis of metformin toxicity.

Although MALA is rare, it is critical to maintain a high index of suspicion in diabetic patients, particularly those with renal impairment or undergoing procedures that may exacerbate kidney injury. Early identification and initiation of extracorporeal treatment are crucial in managing severe metabolic acidosis and improving patient outcomes. This case underscores the importance of careful monitoring and management in diabetic patients with compromised renal function.

## Introduction

Metformin, a biguanide hypoglycemic agent, is the most commonly prescribed oral medication for type 2 diabetes mellitus (T2DM) worldwide [1]. It is considered the first-line treatment for T2DM and a cornerstone in managing nearly all patients with the condition. Metformin has a multitude of actions including: reduced absorption of glucose from the gastrointestinal tract, decreased hepatic gluconeogenesis, and increased peripheral utilization of glucose [2]. 

Metformin-associated lactic acidosis (MALA) is a rare but serious complication, with an estimated prevalence of less than 0.01-0.09 cases/1,000 patient-years [3]. It is most often observed in patients with chronic metformin exposure, particularly those with worsening renal failure, liver disease, or heart failure, though acute intentional overdose can also result in fatal lactic acidosis [3,4]. In critically ill patients, MALA is linked to a mortality rate of over 30%, with shock and the severity of lactic acidosis being key factors that influence the likelihood of death [5]. Management primarily involves supportive care including early recognition and prompt discontinuation of metformin, reversal of metabolic acidosis, and clearance of metformin metabolites through renal replacement therapies, such as intermittent hemodialysis (HD) or continuous renal replacement therapy (CRRT) [6-8].

Here, we present a case of MALA in an elderly female with worsening renal function, who initially presented with symptoms suggesting an acute abdomen.

## Case presentation

A 72-year-old woman with a medical history of T2DM and hypertension presented with a three-day history of abdominal pain, persistent nausea, vomiting, and non-bloody diarrhea. Five days before admission, she underwent an outpatient colonoscopy, which was prepped with polyethylene glycol (PEG) and a sodium phosphate enema. Following the procedure, her oral intake decreased, but she continued her prescribed medications for diabetes and hypertension. On the second day after the colonoscopy, she developed severe diffuse abdominal pain, nausea, non-bloody vomiting, and diarrhea, along with shortness of breath and lightheadedness. She denied any fever, chills, or chest pain. Her home medications included sitagliptin/metformin (Janumet 50-1,000 mg) once daily, amlodipine, atenolol, and a combination of hydrochlorothiazide and losartan. She was a non-smoker, with no history of alcohol or illicit drug use, and had no significant surgical history.

Upon arrival at the emergency department, her vital signs were as follows: temperature 95.6°F, pulse 54 beats per minute, respiratory rate 17 breaths per minute, blood pressure 83/48 mmHg, and oxygen saturation 99% on room air. On physical examination, she appeared lethargic and was tachypneic with increased work of breathing. Abdominal examination revealed diffuse tenderness and pain out of proportion to physical findings but without guarding or rigidity.

**Figure 1:**
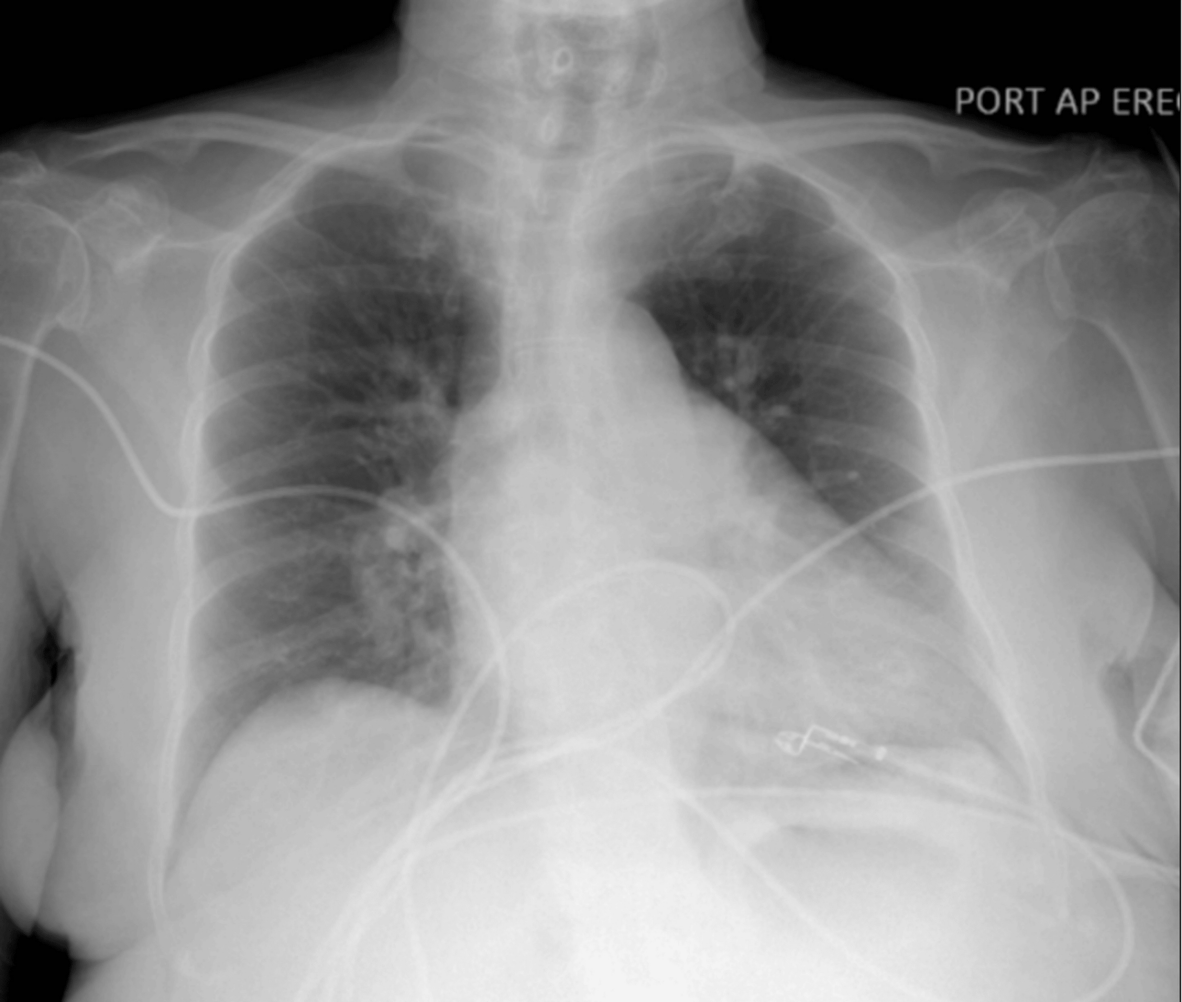
CXR showing mild bilateral vascular congestion. No gas under the diaphragm was noted CXR: Chest X-ray

Initial laboratory findings (Table 1) showed elevated blood urea nitrogen (BUN) and creatinine. Blood gas showed severe lactic acidosis with pH 6.77, bicarbonate 4, and lactic acidosis 6.70 (Table 2). A chest X-ray (CXR) did not reveal pneumoperitoneum but showed mild bilateral vascular congestion (Figure 1). The patient’s condition continued to worsen, with blood pressure remaining low despite 2.5 L of intravenous fluid and 4 ampules of sodium bicarbonate. The refractory acidosis and hypotension led to the initiation of low-dose norepinephrine to support blood pressure and perfusion. Given her abdominal pain, severe acidosis, and recent colonoscopy, colonic perforation was considered a leading diagnosis. Other potential causes included mesenteric ischemia, septic shock, cardiogenic shock, liver failure, toxic ingestion (e.g., alcohol), and medication-induced lactic acidosis. After consulting with the general surgery team, the decision was made to proceed with an exploratory laparotomy due to her worsening clinical status and abdominal pain disproportionate to physical findings. During the laparotomy, the colon and small bowel were thoroughly examined, but no abnormalities were found. Mild edema in the retroperitoneum was noted, but there was no evidence of purulence.

**Table 1:** Initial pertinent laboratory findings on admission

	Values	Normal Range
White blood count (k/microL)	16.8	4-11
Hemoglobin (gm/dL)	10.9	12-15
Hematocrit (%)	35.2	36-46
Bicarbonate (mEq/L)	4.2	22-29
Anion gap (mEq/L)	41	9-18
Blood urea nitrogen (BUN) (mEQ/L)	113	9.8-20.1
Creatinine (mg/dL)	9.9	0.57-1.11
Estimated glomerular filtration rate (eGFR) (mL/min)	4	>60
Aspartate aminotransferase (AST) (units/L)	453	5-34
Alanine aminotransferase (ALT) (units/L)	579	0-55
Blood glucose (mg/dL)	98	74-100
Beta hydroxybutyrate (mmol)	9.13	0.02-0.3

**Table 2:** Blood gas values during the first few hours of admission

	pH	PCO_2_	HCO_3_	Lactic Acid (mmol/L)
Admission	6.77	36	4.2	6.70
3 hours	7.23	37	8.8	5.90
5 hours	7.34	11	9.4	6.70

Post-operatively, the patient was transferred to the ICU, where she remained intubated but required minimal ventilator support. With no obvious bowel ischemia or perforation, the differential diagnosis shifted towards metformin toxicity or a possible adverse effect from bowel preparation. Metformin was stopped and CRRT was initiated for acute kidney injury and severe metabolic acidosis. Metformin levels were sent for testing, but the results took several days to return.

Following CRRT, the patient's condition improved dramatically, with blood gas values showing marked improvement the following day. Her blood gas results on the next day were as follows: pH 7.42, PCO_2_ 30, PO2 86, HCO_3_ 19, and lactic acid 3.7. Empiric antibiotic therapy with vancomycin and Zosyn was initiated for suspected septic shock but was discontinued once blood cultures were negative. Over the next several days, the patient’s condition continued to improve. She was extubated and eventually transferred out of the ICU. Her serum creatinine decreased to 2.4 mg/dL, lactic acid decreased to 1.4 mmol/L, and blood gases showed sustained correction of acidosis. She was producing more than 1.5 L of urine per day before discharge.

The metformin level, which was finalized a week later, was found to be elevated at 15 mg/L (therapeutic range: 1-2 mg/L, levels >5 mg/L are considered toxic). As a result, metformin was discontinued at discharge, and alternative medications were prescribed for diabetes management.

## Discussion

The history of biguanides to treat diabetes mellitus goes back to medieval Europe for the use of Galega officinalis (French lilac) which contains biguanide as an active component [2]. In the 1920s, guanidine was used in various antidiabetic compounds, and metformin and phenformin, the two main biguanides, were introduced in the late 1950s [2,9,10]. However, metformin was not used regularly when first introduced as it was less potent than other glucose-lowering biguanides (phenformin and buformin), which were discontinued in the late 1970s due to the high risk of lactic acidosis [11]. Metformin has been available in the United States for the treatment of diabetes mellitus since 1995 [2,9]. Since then it has been used as the cornerstone for the management of T2DM because of its low cost and lack of hypoglycemic effects compared to sulfonylureas and other medications [1,11].

In general, metformin works by reducing the absorption of glucose from the gastrointestinal tract, decreasing hepatic gluconeogenesis, and increasing peripheral utilization of glucose [2]. Multiple theories have been proposed on molecular mechanism of metformin that includes inhibition of the mitochondrial respiratory chain, activation of adenosine monophosphate-activated protein kinase (AMPK), inhibition of glucagon-induced elevation of cyclic AMP (cAMP) with reduced activation of protein kinase A (PKA), inhibition of mitochondrial glycerophosphate dehydrogenase, and an effect on gut microbiota [12,13].

Some of the commonly reported side effects of metformin include nausea, vomiting, stomach upset, diarrhea, weakness, or a metallic taste associated with it [14]. MALA is a relatively rare complication of metformin. The estimated prevalence of MALA is less than 0.01-0.09 cases/1,000 patient-years [3]. Impaired kidney function is the most important risk factor for MALA. The metformin undergoes renal excretion and has a mean plasma elimination half-life after oral administration of between 4.0 and 8.7 hours [15]. This elimination is prolonged in patients with renal impairment and correlates with creatinine clearance. This is the main reason to monitor renal function in patients taking metformin. Therapeutic levels of metformin range from 0.5 to 1.0 mg/L in the fasting state and 1 to 2 mg/L after a meal [15]. Metformin levels of more than 5 mg/L have been associated with profound lactic acidosis and are considered to be toxic levels. Confirmation of metformin toxicity requires measurement of serum metformin by high-performance liquid chromatography-tandem mass spectrometry [16]. 

The American Diabetes Association and European Association for the Study of Diabetes report that metformin seems safe unless the estimated glomerular filtration rate (eGFR) falls to <30 mL/min/1.73m^2^ [17]. The United States Food and Drug Association guidelines were changed in lieu in 2016 stating that metformin is contraindicated in eGFR of less than 30 mL/min and also recommended not to start metformin if eGFR is between 30-45 mL/min [18]. The association of MALA in patients with stable chronic kidney disease (CKD) has not been well established. A meta-analysis done in 2014 did not support a strong association of MALA with metformin in patients with stable CKD [19]. However, patients with significant renal impairment, either due to advanced CKD or due to acute kidney injury, remain at high risk for metformin toxicity [20,21]. MALA has been associated with high mortality. Patients requiring critical care have a mortality of greater than 30% [5]. Other causes of high anion gap metabolic acidosis (HAGMA) include septic shock, acid alcohol ingestion, salicylate ingestion, propylene glycol toxicity, and liver failure should be ruled out during the evaluation for possible MALA.

The initial management of MALA in the critical care setting involves aggressive resuscitation, supportive management, and management of comorbid conditions [6,8]. Metformin is an easily dialyzable medication due to its small molecular weight and its lack of protein binding [8,22]. Management of MALA not responding to resuscitative measures includes prompt initiation of intermittent HD or CRRT. Intermittent HD is superior in clearing lactate when compared with CRRT [8]. Studies suggest initiating dialysis when lactate concentration is >15 mol/L, pH is <7.0 with shock, or if it is resistant to other medical therapy. However, the indications are not clear-cut and depend on the presentation of each individual [8,22]. 

Our patient had poor oral intake for several days for bowel preparation, causing dehydration. In addition, she got PEG and sodium phosphate enema. She continued to take metformin in the setting of worsening renal function which brewed a perfect storm to create MALA. She presented with a lactate of 6 mmol/L, pH of 6.77, and also failed to respond to the use of other medical measures (aggressive resuscitation, bicarbonate). She showed a dramatic improvement in her acidosis after the initiation of CRRT. High clinical suspicion for the possibility of metformin toxicity and the prompt initiation of CRRT is of utmost importance especially if the patient fails to improve with resuscitation and other therapies.

## Conclusions

MALA, though rare, can manifest in critical care settings as persistent metabolic acidosis that is resistant to resuscitation, accompanied by significantly elevated lactate levels. It typically occurs in patients with impaired renal function, making early identification essential. Diagnosis can be particularly challenging when patients present with sepsis, as it shares similar symptoms like severe diffuse abdominal pain as in our patient with other conditions such as gastrointestinal perforation and ischemia. In such cases, especially when the patient has undergone recent procedures like colonoscopy, it is crucial to rule out these differentials. Measurement of metformin levels should be a priority in all suspected cases. This case emphasizes the need to temporarily discontinue metformin in patients who are at risk for dehydration or renal injury, such as those undergoing imaging with intravenous contrast or bowel preparation for procedures like colonoscopy. It also underscores the importance of maintaining a high clinical suspicion in diabetic patients with a history of CKD who present with metabolic acidosis. This is particularly critical when the patient continues to take metformin despite worsening renal function and undergoing procedures that may exacerbate kidney injury. Additionally, the prompt initiation of extracorporeal treatment, such as HD or CRRT, is crucial when indicated to prevent further complications.
